# Diet in Cholera

**Published:** 1849-10

**Authors:** W. R. Handy

**Affiliations:** Baltimore


*Diet in Cholera.* By W. R. Handy, M. D., Baltimore.

It is not our intention to say anything of the origin, pathology, or treatment of this disease. Volumes have been written upon it, which, instead of dispersing the gloom that enshrouds this most fatal epidemic, only serve, it would seem, by the accumulation of conflicting statements, to hide its true character in still greater darkness, and render its mystery thereby, if possible, more mysterious.

All that we propose on the present occasion is, to invite attention to a single point, viz: *the exclusive diet,* prescribed by many physicians, in different sections of our country, and which has been the subject of municipal discussion and regulation in several of our cities, during the prevalence, and in anticipation, of the cholera. The diet referred to, is the exclusion of all *vegetables*, as commonly understood and used under this head, and *fruits* And among the articles to be chiefly employed, rice seems to claim the highest commendation.

The ground for this recommendatory diet is based upon the belief that vegetables and fruits are among the most active *exciting causes* of cholera; and that their use, in fact, establishes the *predisposition*, and thus may be fairly charged with giving existence to the disease.

Now, with all due deference, we respectfully ask if such advice is not in conflict with the laws of a sound physiology and hygiene? and further, in conflict with observations and facts fairly interpreted?

It is to these queries our remarks will be principally confined. And first in reference to the law itself.

M. Magendie, from the many experiments which he instituted, lays it down as " an important hygienic precept," clearly demonstrated, that man requires " a variety of articles of diet,"—that in his food he is omnivorous; and if confined to any one article, however nutritious, that he would loathe, sicken, languish; and, in the cases where he could extend his experiments, as on the lower animals, that they would invariably die,—and that from starvation.

" Variety in food," says Chomel, in his General Pathology, " is necessary to man." " The exclusive use," he continues, " of any one article of food, in those whose unrestrained habits would have been opposed to such a course, almost always terminates in disease."

In speaking of the nutritive properties of proximate (organic) principles, Dr. Bell says, in his work on regimen and longevity, " Health could not be maintained in the exclusive use of any one, but on the contrary, variety is indispensably necessary."

Again. Man's organization seems to demonstrate most conclusively that variety of food is his natural diet. In the language of Dr. Carpenter, in his Physiology, "The construction of his digestive apparatus, as well as his own instinctive properties, point to a mixed diet, as that which is best suited to his wants."

It is well known that the teeth of man combine those, both of the carnivorous and herbivorous animals, having the cutting and rending of the former, as well as the grinding of the latter; and that his alimentary tube holds an intermediate position, between those of both classes of animals; not having the shortness of the carnivorous, nor the complexity of the herbivorous, but combining the digestive capabilities of both, and consequently requiring the food of each.

"Repeated analysis," says Prof. Jackson, in his paper on tea and coffee, in the July No. of the American Journal of the Medical Sciences, "shows that these articles are unfit for nutrition, and has demonstrated that of the aliment that is adapted to healthy nutrition, one-eighth part only consists of albumen, or protein compounds. And whatever is devoid of those substances, cannot perform the office of food, or be fitted for nutrition."

Now protein is the representative of the azotized or albuminous portion of the food, and according to organic chemistry, consists of the following elements, to wit: of carbon forty, hydrogen thirty, nitrogen five, and oxygen twelve; while of the balance of the food, which is pronounced necessary to healthy nutrition, which constitutes by far the largest portion, not less than seven-eighths consist of the non-azotized, non-albuminous, or those articles of food which are deprived of nitrogen, and include, in great measure, the various vegetables and fruits which are excluded as improper diet during the prevalence of cholera.

From all this it would seem clearly to follow, that *variety of food* is most in accordance with, and necessary to fulfil, the physiological or natural law of man's being.

But it may here be objected that vegetables and fruits do not contain any positive elements of nutrition, as they are destitute of nitrogen, and consequently of protein, which latter is regarded according to the degree of its presence, as the proper measure of the comparative nutrient powers of the different kinds of food; it is then said that this protein being entirely absent in the excluded articles of diet, and that they thereby having no nutrient properties, nothing can be lost by their exclusion; and that so far as the nutrition of the being is concerned, that they are of little consequence.

To this we reply, that the position here taken seems more hypothetical than real; more in conflict with standard authorities and facts, than in accordance with truth; for it is not denied that a large class of animals subsist and are nourished solely by the grasses and fruits, and possess the highest amount of energy and strength. And further, that their constitutions are charged with the element nitrogen; and that their tissues, equally with those of the carnivorous, are possessed of the protein compounds; hence, so far as the fact is concerned, both classes of animals are on an equality, as both have the protein in their composition, the only difference being in the manner of obtaining it. The class that live on flesh, take the nitrogen along with their food; while those that live on grass and vegetables, receive their nitrogen along with their respiration, which, combining with the elements of their food, form the protein compounds; so that, admitting protein and the albuminous compounds to constitute the proper elements of nutrition, the herbivorous class of animals derive the same advantage from its presence as the carnivorous.

But admitting that vegetables and fruits possess no nutrient properties for man, even if they do for some of the lower animals, still we think it may be maintained that they are necessary as articles of diet. Every one admits that heat, or a proper amount of *temperature*, is as necessary to life as food; in fact, constitutes one of the vital stimuli. Now it is ascertained that one of the great sources of animal heat is from vegetables and fruit, as well as all of the non-azotized articles of diet; and that this heat is produced by the carbon and hydrogen which are eliminated during the digestion and decomposition of the vegetable diet, combining with oxygen in the production of carbonic acid and water.

On this point, Prof. Jackson asserts that "fatty, starchy and saccharine matters are intended solely for the purpose of calorification, by their combustion or combination with oxygen introduced into the blood by the processes of respiration;" though he does not believe that they are in any way designed for nutrition.

So that allowing that vegetables and fruits contain no nutriment, the fact of their furnishing so much of the heat essential to life, must make them an important, and one would think, an indispensable part of diet; so much so, indeed, that that emergency requiring their expulsion, we would suppose, should be one of the last extreme, arising not under the authority of a hygienic law, for that we have seen advocates and demands their use; but under that of a pathological law, when disease is actually present, and medical treatment demanded. In this latter case we acquiesce, and acknowledge the justice and propriety of the exclusion.

In the herbivorous class, where perspiration is very great, the temperature, by this refrigerating process, is constantly being lowered, and hence, their diet from being exclusively vegetable, gives them an abundant source of supply for the waste of this fundamental element of their existence. In the carnivorous animals "the temperature," says Dr. Carpenter, "appears to be sufficiently kept up by the combustion of the carbon and hydrogen set free by the decay of their tissues."

Man, from the variety of his food, enjoys the capabilities of both classes, and eliminates heat doubtless from both sources.

But the advocates of exclusive diet may exclaim, that even admitting all that has been said about the necessity of variety of diet,—which under ordinary circumstances they acknowledge is all right and proper,—the point at issue, in all that has been advanced, has not been touched:—that what they mean to say is, that the cholera influence is abroad over the land, predisposing the system to an attack, and that vegetables and fruits are among the most prominent causes exciting it into action or producing the disease; hence they assert the propriety and necessity of the advice which excludes all such articles of diet while the cholera prevails.

To this we reply, that cholera is well known to have prevailed as extensively and fatally in the depth of winter, when fruits were scarcely at all to be had, as in the summer, when they are so abundant. And where a case of cholera has occurred after eating fruit, in the language of Dr. Dunglison, it is a mere "coincidence," and such coincidences, in a period of alarm, have been sufficient to excite a terror against its use. And the same author continues, "There is, in truth, not the least reason for presuming, that ripe fruits had any thing more to do with the causation of cholera than any other kind of diet; and how easy it might have been to excite equal prejudices, on no more foundations, against any of the common aliments." The potato is allowed as an article of diet, yet, according to the experiments of Beaumont, whether baked, roasted, or boiled, it is not so digestible as the ripe mellow apple.

Now, according to the pathology of Chomel, there is a marked difference between *predisposing causes* and *predisposition ;* for it must be admitted, that in those places where cholera was most violent and fatally destructive, all had not alike the same predisposition to an attack, and in some there was no predisposition at all, and in such consequently there was no cholera. But had the predisposition been present, then fear, fatigue, anxiety, and a variety of alimentary articles, any or all of them, would have proved equally active exciting causes with fruits in producing the disease.

When diarrhœa or irritation of the intestinal tube is present, we acknowledge the propriety of excluding, as a remedial means, the vegetable diet and fruits. But where this tube is in perfect health, and all the rest of the organs are in a like condition, we cannot see the wisdom or utility of the advice, which refuses the demands of nature by denying that kind of diet which her instinctive teachings in the physiological state the experience of every one abundantly proves to be the surest guides to the preservation of health. In fact, we think it may be safely asserted, that there is no more absolute unity and fixed standard of health, than there is an absolute unity and fixed standard of disease; that consequently there cannot be a fixed regimen of diet suited to every person, any more than there can be one fixed remedy for every disease. For health, which is a generic term, implies the normal action of each and all the organs, whether separately or collectively, as we speak of the health of individual organs, and the general health of the whole. And this normal action may present as various shades of modification as in the various individuals possessing it, and yet all be justly said to be in the possession of health ; hence, with such modifications in the normal action of the organs of different individuals, it does not seem to be any marvel, that different persons should choose different kinds of food, and yet that each different kind of food should nevertheless be the proper kind to the individual so choosing, as all his organs are in a state of health, and consequently cannot err in their choice, and that the difference in choice must be explained by the difference in the shade of their several normal actions in health.

If these positions be established, an *exclusive diet*, then, to ward off disease, where no predisposition exists, we think may not only be seriously questioned, but further honestly stated to be positively injurious. The extent of this injury may be briefly stated under these heads, viz.:

1. By depriving the system of its usual *variety* of food, we thereby injure it by robbing it in a proportionate degree of its otherwise healthy nutrition and proper amount of temperature.

2. By proclaiming this exclusive diet as indispensable, an element of fear is thus introduced, which seizes upon the people, creating such an alarm that they feel at a loss as to what they should eat; reasoning with themselves that probably the doctors may be mistaken about the safety of this and that article, as our neighbors here and there have died after eating it, and that therefore, to be on the safe side, we shall abstain from every such thing as much as possible. Such cases are no fiction; they have occurred; such individuals have abstained, and the consequence has been, as might have been expected, even on common sense reasoning, that their general strength has given way; all the powers of their systems have become weakened, and thus a predisposition, or a standing invitation for cholera or any other disease that may be prevalent, has been created. Fear alone, aside from starvation, is sufficient to do this.

The third and last *injury* we have to mention as arising from this exclusive system of diet, is that inflicted upon a very large and important class of our fellow citizens who supply our markets with those excluded articles, and depend in a great measure on their sale as a means of livelihood for themselves and families.

We say, if the positions taken are tenable, it would seem to follow as a necessary consequence, though not at all intended, that such exclusion is armed with the highest cruelty and oppression on the one hand, as well as with unnecessary deprivations on the other, in resisting the use, of what we all would consider under other circumstances, as most delicious as well as harmless articles of indulgence.

